# Supplementary material for: The interactome of intact mitochondria by cross-linking mass spectrometry provides evidence for coexisting respiratory supercomplexes
Source: Mol Cell Proteomics. 2017 Dec 8;17(2):216–32. doi: 10.1074/mcp.RA117.000470 (PMC5795388; doi:10.1074/mcp.RA117.000470)
Supplement: Supplemental Data [file supp_RA117.000470_133922_0_supp_23978_fzffwf.zip › spectra_annotation/mito_DR_spectra_annotation/500-1-7-1-22-1.pdf]

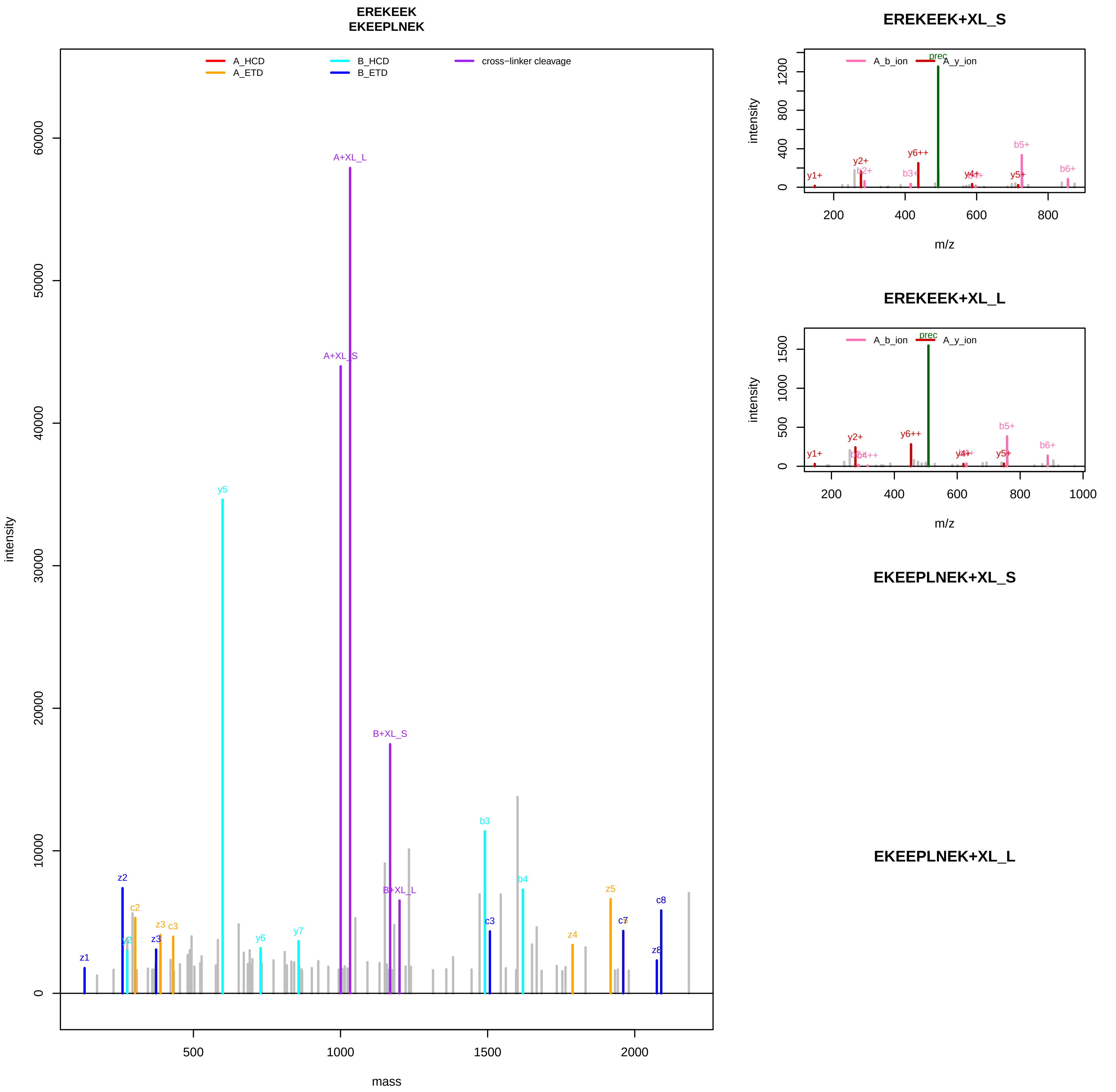
EREKEEK
EKEEPLNEK
A_HCD
A_ETD
B_HCD
B_ETD
cross−linker cleavage
intensity
mass
A+XL_L
A+XL_S
B+XL_S
B+XL_L
y5
y6
y7
b3
b4
z1
z2
z3
c2
z3
c3
c3
z4
z5
c7
z8
c8
EREKEEK+XL_S
A_b_ion
A_y_ion
prec
y1+
y2+
b2+
b3+
y6++
y4+
y5+
b5+
b6+
m/z
EREKEEK+XL_L
EKEEPLNEK+XL_S
EKEEPLNEK+XL_L
